# Synchronous Lymphomas: An Uncommon Affair

**DOI:** 10.4274/tjh.galenos.2019.2019.0187

**Published:** 2020-08-28

**Authors:** Manohor Vidhya

**Affiliations:** 1 Sri Shankara Cancer Hospital and Research Centre, Division of Histopathology, Bangalore, India

**Keywords:** Composite lymphoma, Hodgkin’s lymphoma, Non- Hodgkin lymphoma

An octogenarian man presented with painless left cervical lymphadenopathy. His past history was insignificant with only a mild reduction in platelets in his recent health check.

An excision biopsy of the lymph node revealed a dual population of monotonous small cells with scattered mononucleate and binucleate Reed-Sternberg (RS) cells. Immunostaining confirmed the small cells to be of B-cell phenotype co-expressing CD5 and CD23 and the large cells were CD30+ and CD15+ with weak PAX5 and negative CD20. Epstein-Barr virus results were inconclusive; it was reported as negative as there was a weak and scattered staining pattern and a repeat test was negative. A diagnosis of composite lymphoma, B-small lymphocytic lymphoma with Hodgkin’s lymphoma (HL) was suggested. The bone marrow biopsy revealed involvement of small cell lymphoma (SLL).

Synchronous lymphomas are rare tumors composed of two or more lymphomas in the same tissue site that are morphologically and immunophenotypically distinct. NHL and HL represent mutually exclusive entities [1].

HL has been described in patients with chronic lymphocytic leukemia, a variant of Richter syndrome with an incidence of <0.5% [2,3,4], and following immunosuppressive therapy [5]. Our patient presented with two separate immunohistomorphologies. In the series reported by Xiao et al. [6], although the 2 subtypes showed biological differences with clonality studies, the overall clinical risk and significance did not differ. Whether this is a case of a de novo composite lymphoma or a Richter-like variant transformation of a hitherto undetected SLL is unclear, and the patient refused treatment at diagnosis and was lost to follow-up.

## Figures and Tables

**Figure 1:**
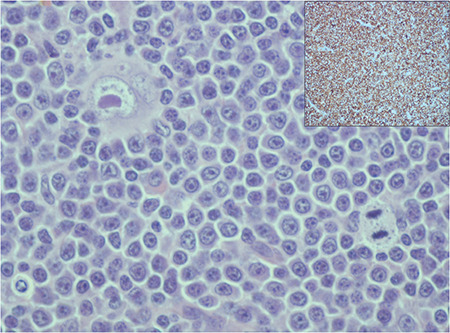
Lymph node showing sheets of monotonous small B cells, positive for CD20 (inset) and scattered large mononucleate Reed-Sternberg cells, with negative staining with CD20 (H&E; 400^x^).

**Figure 2:**
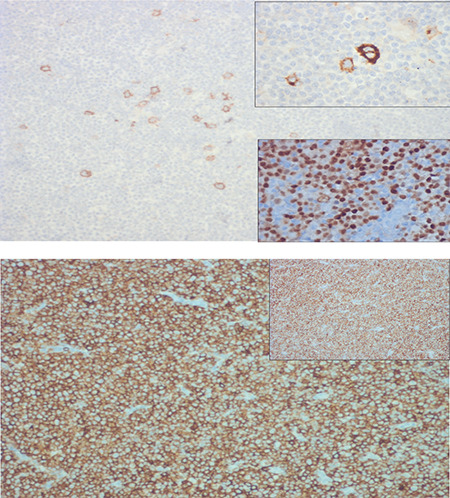
(a) The large cells are CD30+ and CD15+ (inset) (IHC; 100^x^) with weak PAX5 staining (inset arrow). (b) The small cells are CD5+ and CD23+ (inset) (IHC; 100^x^).
